# Correction: The highly efficient removal of HCN over Cu_8_Mn_2_/CeO_2_ catalytic material

**DOI:** 10.1039/d1ra90107a

**Published:** 2021-05-11

**Authors:** Zhihao Yi, Jie Sun, Jigang Li, Yulin Yang, Tian Zhou, Shouping Wei, Anna Zhu

**Affiliations:** Department of Chemistry Defense, Institute of NBC Defense Beijing 102205 China; State Key Laboratory of NBC Protection for Civilian Beijing 102205 China magnsun@tsinghua.edu.cn

## Abstract

Correction for ‘The highly efficient removal of HCN over Cu_8_Mn_2_/CeO_2_ catalytic material’ by Zhihao Yi *et al.*, *RSC Adv.*, 2021, **11**, 8886–8896. DOI: 10.1039/D0RA10177J

The authors regret that in the original manuscript, the author affiliations corresponding to the letters a and b were the wrong way round, meaning the authors were associated with the wrong affiliations.

The correct affiliation information is as given in this correction notice.

The Royal Society of Chemistry apologises for these errors and any consequent inconvenience to authors and readers.

## Supplementary Material

